# Frequencies and Distribution of APOE Gene Polymorphisms and Its Association With Lipid Parameters in the Senegalese Population

**DOI:** 10.7759/cureus.24063

**Published:** 2022-04-12

**Authors:** Maïmouna Touré, Niokhor N Diouf, Souleymane Thiam, Jean P Diop, Mame S Coly, Arame Mbengue, Fatou B Sar, Abdoulaye Ba, Fatou A Diallo, Abdoulaye Samb

**Affiliations:** 1 Laboratory of Human Physiology and Functional Explorations, Faculty of Medicine, Pharmacy and Odonto-Stomatology (FMPOS) University of Cheikh Anta Diop (UCAD), Dakar, SEN; 2 Biology Laboratory, Grand Yoff General Hospital of Idrissa Pouye, Dakar, SEN; 3 Laboratory of Biochemistry and Molecular Biology, Faculty of Medicine, Pharmacy and Odonto-Stomatology (FMPOS) University of Cheikh Anta Diop (UCAD), Dakar, SEN; 4 Human Genetics Laboratory, Faculty of Medicine, Pharmacy and Odonto-Stomatology (FMPOS) University of Cheikh Anta Diop (UCAD), Dakar, SEN; 5 Laboratory of Human Physiology and Functional Explorations, UFR (Unité de Formation et de Recherche) of Health Sciences of Thies, Thies, SEN; 6 Laboratory of Human Physiology and Functional Explorations, UFR (Unité de Formation et de Recherche) of Health Sciences of Thies, Thiès, SEN

**Keywords:** apolipoprotein e, senegalese, lipids, frequencies, polymorphism

## Abstract

Background

Apolipoprotein E is a multifunctional protein that plays an important role in lipid metabolism. It is encoded by the *APOE* gene. However, *APOE* gene polymorphism has not been very well studied in the Senegalese population. Therefore, we studied allele frequencies, genotype distributions, and the relationship between *APOE *gene polymorphisms and lipid parameters in the Senegalese women population.

Methodology

This study included 110 healthy women aged 35-72 years. The mean age was 49.8 ± 8.1 years. For all subjects, lipid parameters were analyzed from fasting serum, and *APOE *genotypes were identified by PCR-RFLP (polymerase chain reaction-restriction fragment length polymorphism) based analysis.

Results

Variations in the frequencies and distribution of the *APOE *alleles and genotypes were observed (ε3: 47.3%; ε2: 43.2%; ε4: 9.6%; and ε2/ε3: 70%; ε2/ε4: 16.4%; ε3/ε3: 10.9%; ε2/ε4: 2.7%). Compared to the ε3ε3 genotype carriers, carriers of the ε3ε4 genotype had a significantly higher rate of total cholesterol (p=0.03) and no high-density lipoprotein-cholesterol (p=0.02). Univariate analysis showed that the *APOE *ε4 allele increases the low-density lipoprotein-cholesterol rate (OR=3.06; 95% CI: 1.16-8.22; p=0.02).

Conclusion

Our study has shown a difference in *APOE* allele frequencies and genotype distributions with a total absence of ε2ε2 and ε4ε4 genotypes in a sample of Senegalese women. We also found that* APOE *gene polymorphism might play a role in plasma lipid levels.

## Introduction

Apolipoprotein E (ApoE) is a polymorphic and multifunctional protein that is mainly synthesized by the liver. It is an essential apolipoprotein in plasma [1] and plays a vital role in the transport, metabolism, and digestion of lipoproteins [2-4]. ApoE promotes efficient clearance of circulating lipoproteins and participates in the cellular efflux of cholesterol [5]. ApoE plays an important role in regulating lipoprotein metabolism by regulating the bind of these lipoproteins to specific receptors. It can bind with different molecules, such as low-density lipoprotein-cholesterol (LDL-C), high-density lipoprotein-cholesterol (HDL-C), very low-density lipoprotein-cholesterol (VLDL-C), and chylomicron to participate in the transformation and metabolism of lipoprotein.

Human ApoE is encoded by the *APOE* gene (OMIM: 107741), which is located on chromosome 19q13.32 [6,7]. The *APOE* gene is polymorphic in nature and possesses three alleles, namely, ε2, ε3, and ε4, which can be found in six genotypic combinations, homozygous (ε2/ε2, ε3/ε3, ε4/ε4) or heterozygous (ε2/ε3, ε2/ε4, ε3/ε4) [8,9].

The *APOE* gene regulates ApoE plasma concentration and its binding capability. The three alleles (ε2, ε3, ε4) of the *APOE* gene are respectively responsible for the production of corresponding apolipoprotein E2, apolipoprotein E3, and apolipoprotein E4 plasma isoproteins [10]. Different alleles lead to structural variations in ApoE protein (E2, E3, E4), influence its functions, and contribute to the variation in lipoprotein concentration, including receptor-binding capacity and lipid metabolism.

Therefore, the ε2, ε3, and ε4 genetic variants and their corresponding protein variations (E2, E3, and E4) have been linked to differential risks of dyslipidemia [11]. Dyslipidemia further confers as a risk factor of atherosclerosis, cerebral infarction, diabetes, and hypertension. Preventing dyslipidemia plays an important role in reducing morbi-mortality rate in the world [12]. At present, there are limited studies on the Senegalese populations. Hence, in the present study, we examined the allelic and genotypic frequencies of the *APOE *gene and its role in the lipid parameters in Senegalese women subjects firstly.

## Materials and methods

Study participants and protocol

A total of 110 healthy subjects were included in this study. According to their ethnicity, there were 104 Wolofs, 49 Peulhs, 44 Toucouleurs, 29 Sereres, 15 Diolas, 15 Lebous, 8 Bambaras, 3 Soces, and 19 other ethnicities. The age of the participants in the study was between 35 and 72 years, and the mean age was 49.79 ± 8.10 years. They were recruited at the physiology of the Cheikh Anta Diop University (UCAD, Dakar, Senegal). The subjects were HIV, HBV, and HCV negative.

Inclusion criteria were as follows: ≥ 18 years of age, females, and having not taken lipid-regulating drugs before or having stopped taking the drug for at least three months. Subjects were excluded if they were diagnosed with a disease that affected blood lipid level. Pregnant and breastfeeding women were also excluded.

All procedures were conducted in accordance with the standards of the Declaration of Helsinki. It was reviewed and approved by the Ethics Committee of UCAD (Reference: Protocole 027512018/CERruCAD). All study participants provided signed informed consent.

Lipid analysis

Approximately 5 mL of fasting venous blood samples (at least 12 hours of overnight fasting) was collected into vacutainers from every participant and stored at -80°C in a deep freezer until further analysis.

All biochemistry parameters were analyzed on an automated Abbott device (ARCHITECT i1000SR, Abbott Laboratories, Abbott Park, Seattle, WA, USA) according to the standard laboratory protocol. The serum samples were assayed for blood lipid profiles. On the heparin tube, we measured the following lipids: apolipoprotein A (Apo A) apolipoprotein B (Apo B), total cholesterol (TC), HDL-C, LDL-C, and triglycerides (TG). TC, HDL-C, and TG were measured by the enzymatic method. Apo A and Apo B were measured by enzyme immunology.

LDL-C level was calculated according to the Friedewald equation: LDL-C = TC − HDL-C − TG/5. No-HDL-C level was calculated by subtracting HDL-C value from the TC value: No-HDL-C = TC − HDL-C.

DNA extraction and genomic assay

DNA Extraction

Genomic DNA was extracted from peripheral blood lymphocytes using a commercial kit (REF A1125, Wizard® Genomic DNA Purification kit, Promega Corporation, Madison, WI, USA), as per the manufacturer’s instructions. The DNA concentration was quantified using a NanoDrop 2000™ spectrophotometer (ThermoFisher Scientific, Waltham, MA, USA).

PCR-RFLP based analysis

*APOE* gene was amplified by polymerase chain reaction (PCR) on ThermoCycler (T-personal, Biometra, Jena, Germany). The PCR conditions included initial denaturation phase at 95°C for 5 minutes followed by 35 cycles of denaturation at 95°C for 30 seconds, annealing at 70°C for 20 seconds, extension phase at 72°C for 20 seconds, and the final elongation at 72°C for 10 minutes. PCR amplification was visually confirmed on 1.5% agarose gel electrophoresis.

**Figure 1:**
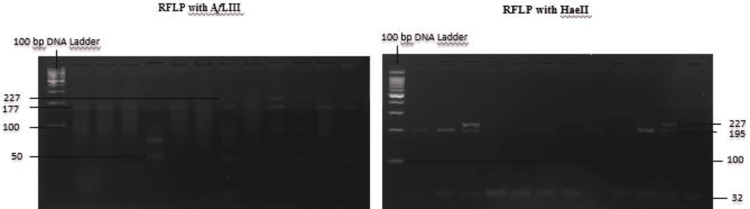
PCR-RFLP gel with AfLIII and HaeII enzyme. ε2/ε2: one band at 227 bp for the HaeII and two bands (at 177 and 50 bp) for AfLIII.  ε3/ε3: two bands (at 195 and 32 bp) for the HaeII and two bands (at 177 and 50 bp) for AfLIII. ε4/ε4: two bands (at 195 and 32 bp) for the HaeII and one band at 227 bp for AfLIII.  ε2/ε3: three bands (at 227, 195, and 32 bp) for the HaeII and two bands (at 177 and 50 bp) for AfLIII.  ε2/ε4: three bands (at 227, 195, and 32 bp) for the HaeII and three bands (at 227, 177, and 50 bp) for the AfLIII.  ε3/ε4: two bands (at 195 and 32 bp) for the HaeII and three bands (at 227, 177, and 50 bp) for AfLIII. PCR-RFLP, polymerase chain reaction-restriction fragment length polymorphism

After confirmation of amplification, 10 µl of each amplified DNA was mixed with each of the two restrictions enzymes (HaeII and AfLIII). The two reactions were allowed to proceed for at least 3 hours at 37˚C. The resulting fragments were analyzed on a 4% agarose gel (Figure 1).

Statistical analysis

The data were expressed as mean ± SD and percentages (%). Allele frequencies were estimated by the gene counting method, and the chi-square test was used to identify departure from Hardy-Weinberg equilibrium. Strength of association between different lipid variables and *APOE* genotypes and alleles was estimated using THE chi-square test and logistic regression. SPSS statistical software Version 23.5 (IBM Corp., Armonk, NY, USA) was used for data analysis. A p-value of ≤0.05 was considered as significant in statistical analysis.

## Results

*APOE* alleles and genotype frequencies

We found that the ε3 allele is the predominant form while the ε2ε3 genotype is the most common form in the study population. In addition, there is a total absence for ε2ε2 and ε4ε4 (Table 1).

**Table 1:** Characteristics of APOE gene polymorphisms VA, allelic variation

Variables	Subjects
Frequency of *APOE* alleles	
Allele n (%)	n = 220 (100)
ε2	95 (43.18)
ε3	104 (47.27)
ε4	21 (9.55)
Distribution of *APOE* genotypes	
Genotype n (%)	n = 110 (100)
ε3ε3	12 (10.91)
ε2ε3	77 (70.00)
ε2ε2	/
ε2ε4	18 (16.36)
ε3ε4	3 (2.73)
ε4ε4	/
Hardy–Weinberg equilibrium	
X²	70.98
VA	0.45
p-value	<0.0001

Comparison of lipid profile between participant subgroups according to *APOE* gene polymorphisms

The ε3ε4 genotype compared to the ε3ε3 genotype had a significantly higher mean level of TC and no-HDL-C (Table 2). We also found that only ε4 carriers had abnormal mean levels of total and LDL-C and that the mean TG level is normal regardless of the genotype considered.

**Table 2:** Difference in the lipid profile of the subjects according to APOE genotype *A p-value of ≤0.05 was considered as significant in statistical analysis using the ANOVA test. The post hoc test was the least significant difference test. Apo A, apolipoprotein A; Apo B, apolipoprotein B; HDL, high-density lipoprotein; LDL, low-density lipoprotein; TC, total cholesterol; TG, triglycerides

Variables	ε3ε3 (a)	ε2ε3 (b)	ε2ε4 (c)	ε3ε4 (d)	a and b	a and c	a and d	b and c	b and d	c and d
	n =12	n = 77	n = 18	n = 3						
TG (g/L)	0.77 ± 0.23	0.83 ± 0.36	0.65 ± 0.21	0.89 ± 0.14	0.59	0.31	0.58	0.04*	0.75	0.24
TC (g/L)	2.02 ± 0.30	2.07 ± 0.46	2.17 ± 0.38	2.65 ± 0.30	0.71	0.37	0.03*	0.41	0.03*	0.08
LDL cholesterol (g/L)	1.30 ± 0.32	1.31 ± 0.42	1.49 ± 0.41	1.80 ± 0.31	0.92	0.21	0.06	0.10	0.04*	0.22
HDL cholesterol (g/L)	0.61 ± 0.12	0.60 ± 0.16	0.59 ± 0.16	0.67 ± 0.05	0.86	0.69	0.53	0.71	0.44	0.38
No HDL cholesterol (g/L)	1.41 ± 0.27	1.47 ± 0.38	1.58 ± 0.36	1.98 ± 0.30	0.61	0.22	0.02*	0.26	0.02*	0.09
TC/HDL	3.41 ± 0.70	3.56 ± 0.78	3.96 ± 1.17	3.95 ± 0.43	0.56	0.08	0.32	0.08	0.43	1.00
LDL/HDL	2.21 ± 0.75	2.29 ± 0.86	2.72 ± 0.95	2.69 ± 0.46	0.78	0.11	0.40	0.05*	0.43	0.95
TG/HDL	1.35 ± 0.65	1.40 ± 0.80	0.97 ± 0.55	0.25 ± 0.14	0.81	0.18	0.98	0.39	0.97	0.75
ApoB/ApoA	0.57 ± 0.13	0.64 ± 0.23	0.66 ± 0.18	0.83 ± 0.32	0.29	0.29	0.07	0.80	0.16	0.22

We also found that carriers of the ε4 allele had higher blood LDL-C levels (Table 3).

**Table 3:** Associations between lipid parameters and APOE allele (categories of lipid parameters are normal and abnormal rates of each parameter) *A p-value of ≤0.05 was considered as significant in statistical analysis using chi-square and odds ratio tests. Apo A, apolipoprotein A; Apo B, apolipoprotein B; HDL, high-density lipoprotein; LDL, low-density lipoprotein

Variables	ε2: chi²; p-value; OR (95%CI)	ε3: chi²; p-value; OR (95%CI)	ε4: chic²; p-value; OR (95%CI)
Total cholesterol (g/L)	0.56; 0.81; 1.14 (0.38–3.48)	2.09; 0.15; 0.43 (0.13–1.39)	3.69; 0.06; 3.02 (0.94–9.72)
HDL cholesterol (g/L)	1.70; 0.19; 2.72 (0.58–12.83)	0.003; 0.96; 0.97 (0.31–3.00)	0.16; 0.69; 0.80 (0.27–2.42)
LDL cholesterol (g/L)	0.03; 0.85; 1.12 (0.35–3.53)	3.80; 0.05*; 0.37 (0.13–1.03)	5.34; 0.02*; 3.06 (1.16–8.22)
Triglycerides (g/L)	0.69; 0.41; 1.05 (1.00–1.09)	0.75; 0.39; 0.95 (0.91–1.00)	0.92; 0.34; 0.95 (0.91–1.00)
No HDL cholesterol (g/L)	0.46 0.50 0.63 (0.17–2.41)	2.83; 0.09; 0.29 (0.06–1.33)	4.12; 0.04*; 4.36 (0.95–20.02)
Total cholesterol/HDL cholesterol	0.36; 0.55; 1.51 (0.39–5.77)	3.62; 0.06; 0.37 (0.13–1.06)	3.64; 0.06; 2.59 (0.95–7.02)
LDL cholesterol/HDL cholesterol	0.32; 0.57; 1.02 (0.99–1.05)	0.40; 0.53; 1.02 (0.99–1.05)	0.48; 0.49; 0.98 (0.95–1.01)
Triglycerides/HDL cholesterol	0.49; 0.49; 1.05 (0.99–1.09)	0.60; 0.44; 1.03 (1.00–1.07)	0.73; 0.39; 0.97 (0.93–1.01)
ApoB/ApoA	1.02; 0.31; 2.20 (0.46–10.45)	1.12; 0.29; 0.56 (0.19–1.67)	1.35; 0.25; 1.84 (0.65–5.21)

## Discussion

ApoE, one of the major protein components of lipoproteins, plays an essential role in the circulation and the metabolism of blood lipids [13]. It is encoded by a gene that is characterized by polymorphisms. At present, there are limited studies on *APOE* gene polymorphism in the Senegalese population. Correlation between allelic and genotypic frequencies of *APOE* polymorphisms versus the risk of dyslipidemia served as a key attraction to conduct this study.

In this study, the analysis showed the three alleles of the *APOE* gene, which are all present in the Senegalese population with frequency distribution as ε3(47.3%)>ε2(43.2%)>ε4(9.6%). Furthermore, It should be mentioned that the *APOE* genotype distribution in this study was ε2ε3(70%)>ε2ε4(16.4%)>ε3ε3(10.9%)>ε3ε4(2.7%). However, some genotypes such as ε2ε2 and ε4ε4 were totally absent in our study population of adult subjects. We estimate that the absence of the ε2ε2 and ε4ε4 genotypes, in our study population consisting of elderly subjects, would be justified by a relatively short life expectancy for individuals harboring these genotypes. In effect, ε2ε2 homozygosity can precipitate type III hyperlipoproteinemia [14] while ε4ε4 would increase plasma LDL levels and the risk for atherosclerosis [2]. This differs from the results of some studies conducted in other populations such as in Algeria [15], where all *APOE* genotypes were present, and in another study from Saudi Arabia, researchers noted a total absence of ε2 and consequently the absence of the corresponding genotypes ε2ε3, ε2ε4, and ε2ε2 [16].

In the study, *APOE* ε2ε3 is the most common genotype and ε3 is the most predominant allele. This is contrary to previous studies because the ε3 allele is generally the most predominant allele but the most common genotype was ε3ε3 [17,18], and the worldwide frequency of the ε2, ε3, and ε4 alleles is 8.4%, 77.9%, and 13.7%, respectively [19]. Authors have reported that the ε2 isoform is the least common [15], which does not support our results, although the frequencies of *APOE* alleles and genotypes vary considerably between different ethnicities and populations [20].

In our study, we found that individuals harboring ε4 allele has higher blood LDL-cholesterol levels (OR=3.06; 95% CI: 1.16-8.22; p=0.02). In addition, the study shows that the individuals harboring ε3ε4 genotype compared to the individuals harboring ε3ε3 genotype had significantly a higher means levels of TC (p=0.03) and no-HDL-C (p=0.02). Our results are in line with the published studies. In the literature data, the ε4 isoform was associated with an increase in the TC and LDL-cholesterol concentrations when the ε3/ε3 homozygote carriers are used as references. Similar results have been observed in different studies [21,22]. Our study has shown a significant association between ε2 allele and the plasma TG levels (OR=1.05; 95% CI: 1.00-1.09). Several previous studies rather reported increased plasma TG levels in individuals harboring allele ε2 in healthy populations [23-25] and that TG concentration was significantly lower in the carrier of ε3 allele than in the carriers of ε2 or ε4 allele [21].

ApoE isoforms differ in their binding affinity to serum cholesterol and hence in their ability to remove dietary fats from the blood [26]. Accordingly, total serum cholesterol levels differ between *APOE* alleles and genotypes [26,27]. In view of these findings, it appears that despite differences of only one or two amino acids, the structural and functional differences between the three apoE isoforms may have a profound effect on disease risk [28]. The mature form of ApoE has two structural domains separated by a hinge region. The amino-terminal domain (amino acids ~1-191) contains the LDL receptor-binding region, and the carboxyl-terminal domain (amino acids ~225-299) contains the lipid-binding region [2]. The structural basis of the three isoforms occurs through amino acid exchanges of amino acid residues of the polypeptide chain at positions 112 and 158 of the protein sequence where cysteine (Cys) or arginine (Arg) is present. The most common isoform, ApoE3, has a Cys at position 112 and an Arg at position 158 (Cys 112, Arg 158), ApoE2 has a Cys at position 112 and 158 (Cys 112, Cys 158), and ApoE4 has an Arg at positions 112 and 158 (Arg 112, Arg 158) [2]. In ApoE3, Arg-158 forms a salt bridge with aspartic acid-154, whereas in ApoE4, with Cys-158, this salt bridge is interrupted and aspartic acid-154 interacts with Arg-150, modifying the whole region receptor binding [2]. ApoE2 isoform shows defective binding to hepatic lipoprotein receptors [28]. Thus, the differential abilities of ApoE isoforms to bind to hepatic lipid receptors may contribute to ApoE isoform-specific effects in disease. This was confirmed by the study of Eto et al., which showed that the E2 allele and the E4 allele are associated with an increased risk of ischemic heart disease compared to the E3 allele [29].

The limits of our work are imputed to the study population in view of its small number and its exclusive composition of women. This study focused exclusively on a female population. In fact, in the literature, it is demonstrated that obesity is associated with polymorphisms of the *APOE* gene. In view of the variations in the prevalence of obesity according to gender, we wanted to first conduct a study in a female population and then, if possible, in an exclusively male population.

## Conclusions

Our study has shown a significant difference in *APOE* allele frequencies and genotype distributions in the Senegalese women compared to other populations. The total absence of ε2ε2 and ε4ε4 genotypes in this Senegalese adult population requires a resumption to work on a younger cohort to better establish the results. We also found in the study that *APOE* gene polymorphism might play a role in determining plasma lipid levels.

## References

[1] Rebeck GW, LaDu MJ, Estus S, Bu G, Weeber EJ (2006). The generation and function of soluble apoE receptors in the CNS. Mol Neurodegener.

[2] Mahley RW, Rall SC Jr (2000). Apolipoprotein E: far more than a lipid transport protein. Annu Rev Genomics Hum Genet.

[3] Raffaï RL, Hasty AH, Wang Y (2003). Hepatocyte-derived ApoE is more effective than non-hepatocyte-derived ApoE in remnant lipoprotein clearance. J Biol Chem.

[4] Hagberg JM, Wilund KR, Ferrell RE (2000). APO E gene and gene-environment effects on plasma lipoprotein-lipid levels. Physiol Genomics.

[5] Moreno JA, Pérez-Jiménez F, Marín C (2004). The effect of dietary fat on LDL size is influenced by apolipoprotein E genotype in healthy subjects. J Nutr.

[6] Weisgraber KH, Rall SC Jr, Mahley RW (1981). Human E apoprotein heterogeneity. Cysteine-arginine interchanges in the amino acid sequence of the apo-E isoforms. J Biol Chem.

[7] Rall SC Jr, Weisgraber KH, Mahley RW (1982). Human apolipoprotein E. The complete amino acid sequence. J Biol Chem.

[8] Paik YK, Chang DJ, Reardon CA, Davies GE, Mahley RW, Taylor JM (1985). Nucleotide sequence and structure of the human apolipoprotein E gene. Proc Natl Acad Sci U S A.

[9] Lin QY, Du JP, Zhang MY (1999). Effect of apolipoprotein E gene Hha I restricting fragment length polymorphism on serum lipids in cholecystolithiasis. World J Gastroenterol.

[10] Shcherbak NS (2001). Apolipoprotein E gene polymorphism is not a strong risk factor for diabetic nephropathy and retinopathy in type I diabetes: case-control study. BMC Med Genet.

[11] Bennet AM, Di Angelantonio E, Ye Z (2007). Association of apolipoprotein E genotypes with lipid levels and coronary risk. JAMA.

[12] Lewington S, Whitlock G, Clarke R (2007). Blood cholesterol and vascular mortality by age, sex, and blood pressure: a meta-analysis of individual data from 61 prospective studies with 55,000 vascular deaths. Lancet.

[13] Eichner JE, Dunn ST, Perveen G, Thompson DM, Stewart KE, Stroehla BC (2002). Apolipoprotein E polymorphism and cardiovascular disease: a HuGE review. Am J Epidemiol.

[14] Mahley RW (1988). Apolipoprotein E: cholesterol transport protein with expanding role in cell biology. Science.

[15] Boumendjel S, Khodja D, Hamri A, Benlatreche C, Abadi N (2013). [Apolipoprotein E polymorphism and cerebral stroke]. Ann Biol Clin (Paris).

[16] Al-Khedhairy AA (2004). Apolipoprotein E polymorphism in Saudis. Mol Biol Rep.

[17] Yin R, Pan S, Wu J, Lin W, Yang D (2008). Apolipoprotein E gene polymorphism and serum lipid levels in the Guangxi Hei Yi Zhuang and Han populations. Exp Biol Med (Maywood).

[18] Tanyanyiwa DM, Marais AD, Byrnes P, Jones S (2016). The influence of ApoE genotype on the lipid profile and lipoproteins during normal pregnancy in a Southern African population. Afr Health Sci.

[19] Heffernan AL, Chidgey C, Peng P, Masters CL, Roberts BR (2016). The neurobiology and age-related prevalence of the ε4 allele of apolipoprotein E in Alzheimer's disease cohorts. J Mol Neurosci.

[20] Corbo RM, Scacchi R (1999). Apolipoprotein E (APOE) allele distribution in the world. Is APOE*4 a 'thrifty' allele?. Ann Hum Genet.

[21] Wang C, Yan W, Wang H, Zhu J, Chen H (2019). APOE polymorphism is associated with blood lipid and serum uric acid metabolism in hypertension or coronary heart disease in a Chinese population. Pharmacogenomics.

[22] Mahley RW, Weisgraber KH, Huang Y (2009). Apolipoprotein E: structure determines function, from atherosclerosis to Alzheimer's disease to AIDS. J Lipid Res.

[23] Masemola ML, Alberts M, Urdal P (2007). Apolipoprotein E genotypes and their relation to lipid levels in a rural South African population. Scand J Public Health Suppl.

[24] Song Y, Stampfer MJ, Liu S (2004). Meta-analysis: apolipoprotein E genotypes and risk for coronary heart disease. Ann Intern Med.

[25] Jemaa R, Elasmi M, Naouali C (2006). Apolipoprotein E polymorphism in the Tunisian population: frequency and effect on lipid parameters. Clin Biochem.

[26] Davignon J (2005). Apolipoprotein E and atherosclerosis: beyond lipid effect. Arterioscler Thromb Vasc Biol.

[27] Liu CC, Liu CC, Kanekiyo T, Xu H, Bu G (2013). Apolipoprotein E and Alzheimer disease: risk, mechanisms and therapy. Nat Rev Neurol.

[28] Rall SC Jr, Weisgraber KH, Innerarity TL, Mahley RW (1982). Structural basis for receptor binding heterogeneity of apolipoprotein E from type III hyperlipoproteinemic subjects. Proc Natl Acad Sci U S A.

[29] Eto M, Watanabe K, Makino I (1989). Increased frequencies of apolipoprotein epsilon 2 and epsilon 4 alleles in patients with ischemic heart disease. Clin Genet.

